# Diagnostic accuracy of machine-learning-assisted detection for anterior cruciate ligament injury based on magnetic resonance imaging: Protocol for a systematic review and meta-analysis

**DOI:** 10.1097/MD.0000000000018324

**Published:** 2019-12-16

**Authors:** Yongfeng Lao, Bibo Jia, Peilin Yan, Minghao Pan, Xu Hui, Jing Li, Wei Luo, Xingjie Li, Jiani Han, Peijing Yan, Liang Yao

**Affiliations:** a Second Clinical Medical College of Lanzhou University; b Public Health School of Lanzhou University; c Jingtaixian Hospital of traditional Chinese Medicine; d Gansu University of Chinese Medicine; e Institute of Clinical Research and Evidence-Based Medicine, Gansu Provincial Hospital, Lanzhou, China; f Health Research Methodology | Department of Health Research Methods, Evidence and Impact, McMaster University, Canada.

**Keywords:** anterior cruciate ligament, diagnostic test accuracy, magnetic resonance imaging, meta-analysis, protocol, systematic review

## Abstract

**Background:**

Although many machine learning algorithms have been developed to detect anterior cruciate ligament (ACL) injury based on magnetic resonance imaging (MRI), the performance of different algorithms required further investigation. The objectives of this current systematic review are to evaluate the diagnostic accuracy of machine-learning-assisted detection for ACL injury based on MRI and find the current best algorithm.

**Method:**

We will conduct a comprehensive database search for clinical diagnostic tests in PubMed, EMBASE, Cochrane Library, and Web of science without restrictions on publication status and language. The reference lists of the included articles will also be checked to identify additional studies for potential inclusion. Two reviewers will independently review all literature for inclusion and assess their methodological quality using Quality Assessment of Diagnostic Accuracy Studies version 2. Clinical diagnostic tests exploring the efficacy of machine-learning-assisted system for detecting ACL injury based on MRI will be considered for inclusion. Another 2 reviewers will independently extract data from eligible studies based on a pre-designed standardized form. Any disagreements will be resolved by consensus. RevMan 5.3 and Stata SE 12.0 software will be used for data synthesis. If appropriate, we will calculate the summary sensitivity, specificity, positive likelihood ratio, negative likelihood ratio, and diagnostic odds ratio of machine-learning-assisted diagnosis system for ACL injury detection. A hierarchical summary receiver operating characteristic (HSROC) curve will also be plotted, and the area under the ROC curve (AUC) is going to calculated using the bivariate model. If the pooling of results is considered inappropriate, we will present and describe our findings in diagrams and tables and describe them narratively.

**Result:**

This is the first systematic assessment of machine learning system for the detection of ACL injury based on MRI. We predict it will provide highquality synthesis of existing evidence for the diagnostic accuracy of machine-learning-assisted detection for ACL injury and a relatively comprehensive reference for clinical practice and development of interdisciplinary field of artificial intelligence and medicine.

**Conclusion:**

This protocol outlined the significance and methodologically details of a systematic review of machine-learning-assisted detection for ACL injury based on MRI. The ongoing systematic review will provide high-quality synthesis of current evidence of machine learning system for detecting ACL injury.

**Registration:**

The meta-analysis has been prospectively registered in PROSPERO (CRD42019136581).

## Abstract 2

Supplemental Digital Content is available in the text

## 1 Introduction

Anterior cruciate ligament (ACL) injury is a common sports injury, it has a significant effect on knee function which may cause joint instability, decreased activity and poor knee-related quality of life.^[1,2]^ There are approximately 200,000 cases per year in the United States alone.^[3]^ A cohort study found an incidence of ACL tears of 3.2% for men and 3.5% for women during a 4-year period in America.^[4]^ Surgery reconstruction is the predominant treatment for an ACL injury in current practice.^[1]^ Direct hospital costs of ACL reconstruction surgery in 2014–15 were estimated to be $142 million.^[5]^

Timely and accurate diagnosis and treatment of ACL injury could prevent the emergence of cartilage degeneration, the progression of bone contusion, the aggravation of traumatic arthritis or the occurrence of knee joint dysfunction.^[6]^ Arthroscopy is the gold standard for evaluating internal disorders and other lesions of the knee.^[7]^ However, arthroscopy constitutes a relatively expensive and invasive examination which restricted its routine use in clinical practice. As a non-invasive method with good soft tissue contrast, high spatial resolution, multi-parameter and multi-range imaging for the evaluation of knee lesions, magnetic resonance imaging (MRI) has been widely used in the diagnosis of ACL injury with appreciable diagnostic performance when compared with arthroscopy.^[8]^ But it might be sometime tiresome, time-consuming and prone to errors for radiologists to detect various injuries from magnetic resonance (MR) scans and determine the level of injury. Furthermore, making an accurate diagnosis based on MR images may still be challenging for a non-musculoskeletal radiologist, a trainee on call, or a clinician in a rural area without access to subspecialty radiology.

Recently, new information and communication technologies have changed the way of operations in all fields of life such as intelligent transportation systems, agriculture, education, and healthcare systems.^[9]^ Machine learning technology, categorized into supervised machine learning, unsupervised machine learning, semi-supervised machine learning and reinforcement machine learning which can automatically or semi-automatically predict development trends and potential rules of medical data may provide a solution to traditional diagnostic defects because of its important applications for disease diagnosis and medical research.^[9,10]^ Deep learning, a powerful emerging branch of machine learning, has yielded breakthroughs in computer vision benchmarks in recent years.^[11]^ This technology underlies almost all of the most recent advances in artificial intelligence over the past several years, from self-driving cars to voice and facial recognition-tasks which promote researchers to consider its potential applications in the healthcare field.^[12]^ The development of deep learning technology makes it more accurate to analysis medical datasets when compared with other machine learning technology that efforts to apply deep learning methods to health care are already planned or underway.^[13]^

In the past decade, several computer-aided diagnostic systems based on machine learning including deep learning technology had been developed to detect ACL injury automatically or semi-automatically.^[12,14–16]^ However, different diagnostic tests may use different algorithms of which the diagnostic results in validation sets were not always consistent. Furthermore, the limited sample size of the validation sets in original studies may cause confusing results. A high-quality meta-analysis which can pool data from individual studies and reanalyze using established statistical methods has been increasingly regarded as one of the key tools for achieving evidence.^[17–19]^ However, there is not any meta-analysis to synthesize the existing studies on the diagnosis of ACL injury by machine learning for more reliable results.

Therefore, we will conduct this diagnostic test accuracy (DTA) meta-analysis to assess the value of machine-learning-assisted diagnosis for detecting ACL injury based on MRI.

## 2 Method and analysis

### 2.1 Patient and public involvement

There is no patient and public involvement in the whole process when we conduct this research.

### 2.2 Registration and reporting

The protocol of this systematic review and meta-analysis had been prospectively registered at PROSPERO (CRD42019136581) for quality control when we started searching for relative studies.^[20,21]^ Preferred Reporting Items for Systematic Review and Meta-Analysis (PRISMA)^[22]^ will be referenced throughout the study and this protocol is based on an extension of PRISMA for protocol (PRISMA-P).^[23]^

### 2.3 Eligibility criteria

#### 2.3.1 Type of studies

All primary prospective and retrospective clinical diagnostic accuracy studies exploring the diagnostic efficacy of machine-learning-assisted detection for ACL injury with quantitative data will be included. No restriction is set for specific machine learning algorithm initially. There will be no limitations on the year of publication. Only literature published in English will be considered. Furthermore, editorials, letters, and comments et al will not be considered. Relative reviews will be checked to track their references for potentially eligible studies. Studies will be excluded when there is not sufficient data or the full texts could not be obtained.

#### 2.3.2 Participants

Only studies in patients with an ACL injury will be considered for inclusion for this review. There will be no restriction for other comorbidities of the knee joint. ACL injury was confirmed based on different process criteria in different research (ie, visual inspection by a board-certified subspecialist musculoskeletal radiologist,^[12]^ 3 musculoskeletal radiologists established reference standard labels based on an internal validation set of 120 exams^[16]^). Different diagnostic criteria of included studies will all be extracted for later analysis. The knee joint MR images of all participants should be available to the machine learning system and scanned for identification and diagnosis of ACL injury in all included studies.

#### 2.3.3 Index test

The machine-learning-assisted system based on different algorithms was used for detecting ACL injury through MR images in each included study. Some studies compared the diagnostic performance of different algorithms or models,^[12,14]^ then we will consider all the diagnostic data of each algorithm instead of only the best one. As a general process of machine diagnosis system development, it needs to go through training set for model training, turning set for algorithm optimizing, validation set for verifying the diagnostic performance of the final model. We will consider the validation set for diagnostic efficacy evaluation which tends to be the final optimal data. Results from machine detection were compared with a diagnosis from experienced radiologists^[12,14]^ or examined by medical experts^[15]^ et al in primary studies.

#### 2.3.4 Outcomes

Only studies reporting quantitative diagnostic results of machine-learning-assisted detection compared to the reference test such as sensitivity, specificity or the area under the receiver operating characteristic curve (AUC) will be considered for inclusion. We should be able to extract or calculate the true positive, true negative, false positive, false negative of the index test, otherwise, it will be excluded.

### 2.4 Search strategies

We will conduct a comprehensive computer-based literature search without year and language restrictions to identify all relevant clinical diagnostic tests which might improve the quality of retrieval.^[24]^ The key text words of our search strategies are “artificial intelligence”, “machine learning”, “deep learning”, and “anterior cruciate ligament”. The following electronic databases will be searched: PubMed, EMBASE, Cochrane Library, and Web of Science. The format and combination of search terms are adjusted to fit each electronic database. Searching strategies in different databases are presented in supplemental content. Additionally, we will manually retrieve congress reports and conference proceedings. References of included study will also be traced back to find potential qualified studies. Grey literature will be identified through Google Scholar.^[25]^

### 2.5 Study selections

**Figure 1:**
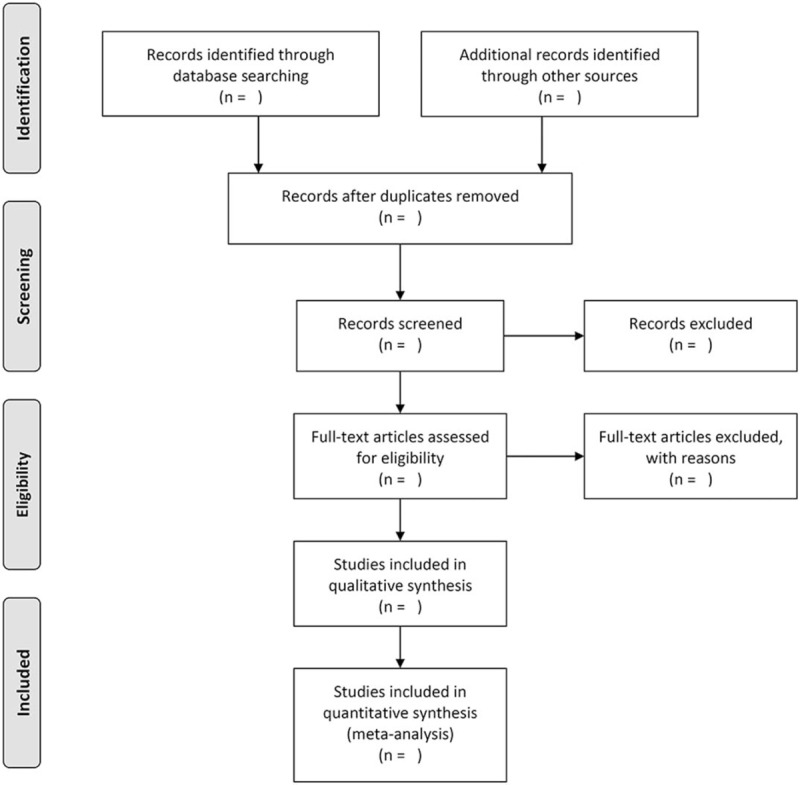
PRISMA flow diagram of studies selection process.

Literature records will be imported into ENDNOTE X7 software for management after literature retrieval. We will exclude duplicates at first, and then 2 reviewers will independently screen the titles and abstracts of all the remaining records for later full-text selection of potentially eligible studies. Final inclusion will be made after checking all the full texts from the previous step while excluded studies and the reasons for their exclusion will be recorded in EXCEL 2016 (Microsoft, Redmond, WA, www.microsoft.com). Any dispute arising in the pairing process will be resolved by consensus. We will try to contact with the main authors if the full text cannot be obtained. The selection process will be presented in a PRISMA flow diagram (see Fig. 1).

### 2.6 Data extraction

Two reviewers will independently search and extract target information in included articles based on standard data extraction form in Excel 2016 designed in advance. The standard data extraction form will contains the basic information of target studies (first author, year of publication, study design, sample size, the gender and age composition of participants, the characteristics of index test and reference test et al) and result data (sensitivity, specificity, true positive, false positive, false negative, true negative et al) of the included studies. When data extraction has been finished separately, the 2 reviewers will check together for a final version. Any disagreements will be resolved by consensus. If the data was not fully reported, we will try to contact the authors of the papers asking for the original data. Studies will be excluded if we could not have access to the necessary data.

### 2.7 Risk of bias assessment

Two reviewers will assess the bias of included studies independently and check together by using the QUADAS-2 tool^[26]^ which comprises 4 domains: patient selection, index test, reference standard, and flow and timing. Each domain can be rated as “High risk”, “Low risk”, or “Unclear risk?” to assess the risk of bias from different angles. Furthermore, the first three domains will be assessed for applicability concerns and rated using the same categories. Any disagreement will be resolved by consensus. Studies with high risk of bias will be considered for exclusion or sensitivity analysis.

### 2.8 Statistical analysis and data synthesis

We will standardize extracted information of each included study at first. For some important general nonnumerical information, we will present it in tables and supplements and describe qualitatively. We will extract or calculate binary diagnostic accuracy data from all studies and constructed 2 × 2 tables for each study. Each sensitivity and specificity with 95% confidence interval (CI) will be presented in forest plots and in the receiver operating characteristics space.

To generate pooled estimates of sensitivity and specificity, we will apply bivariate meta-analysis methods.^[27]^ Review Manager (RevMan; Version 5.3) and Stata SE (Version 12.0) software will be used for data synthesis. Summary measures for diagnostic accuracy of the machine-learning-assisted detection (sensitivity, specificity, diagnostic odds ratios, positive likelihood ratio, negative likelihood ratio) will be calculated using a bivariate random-effects model. A hierarchical summary receiver operating characteristic (HSROC) curve will also be plotted, and the area under the ROC curve (AUC) is going to calculated using the bivariate model. If applicable, we will conduct subgroup based on pre-set criteria to find more information:

(1) partial injury vs complete tear;
(2) with knee joint comorbidity versus without knee joint comorbidity;
(3) different machine learning algorithms used in primary studies;
(4) different MRI sequences and magnet intensities used in primary studies.

If we find that pooling of the results would be inappropriate (for instance, the heterogeneity is too great, or the number of included studies are too small), we will use a narrative approach to synthesize the data.

### 2.9 Heterogeneity investigation

Cochrane *χ*^2^ test and *I*^2^ will be used to quantitatively determine the heterogeneity (test level is α = 0.05). Significant heterogeneity is defined as *P* < .05. The magnitude of heterogeneity can be categorized as low (0%–30%), moderate (30%–50%), considerable (50%–70%) and substantial (70%–100%).^[28]^ To better interpreter the source of heterogeneity, we will conduct exploratory subgroup analysis in addition to the above mentioned if applicable. If data are too heterogeneous to pooling of effect sizes in a meaningful or valid way, we will use a narrative approach to synthesize the data.

### 2.10 Reporting bias and sensitivity analysis

If there are more than 10 studies for data synthesis, we will carry out an informal visual inspection of funnel plots and Egger test to explore the potential publication bias.^[29,30]^ Statistical significance will be considered with respect to a p-value of <0.1 due to the low power of the test. Additionally, sensitivity analysis will be carried out by excluding each study from the overall results and then results will be compared with overall findings to evaluate the stability of the results.

### 2.11 Confidence in cumulative evidence

The Assessment of Multiple Systematic Reviews tool (AMSTAR 2) will be used to assess the methodological quality of finished systematic review.^[31,32]^ And the Grades of Recommendation, Assessment, Development and Evaluation (GRADE) system will be applied for quantifying absolute effects and quality of the evidence.^[33,34]^

### 2.12 Ethics and dissemination

There is no need for a requirement of ethical approval and informed consent for this study because it is based on published literature. And the results of this systematic review will be submitted to a peer-reviewed journal for publication and information sharing.

## 3 Discussion

Computational scientists are trying to develop different machine learning systems based on various algorithms for clinical applications. The use of machine learning in medicine promises to free doctors from repetitive labor and may be able to perform better. Additionally, the introduction of machine learning to health care might have a promoting effect on the development of health system especially in the rural and remote areas,^[35]^ as well as bring to new opportunities and challenges to develop clinical guidelines.^[36,37]^ However, there may be some obstacles to the application of machine learning algorithms in clinical practice. One of them is that models which were familiar to computational scientists with different diagnostic performance are unfamiliar to clinicians. Furthermore, there appears to be some difficulties and limitations according to our previous work that a considerable number of diagnostic tests were based on retrospective case data and may cannot accurately evaluate the clinical diagnosis effect when introduced to the clinic for instance.

This meta-analysis will systematically evaluate the diagnostic efficiency of the machine learning system for ACL injury firstly. We will get the overall sensitivity and specificity of the machine learning system and the overall sensitivity and specificity of the different algorithms. The results hope to provide a state of current research and new research direction of the interdisciplinary field of artificial intelligence and medicine for ACL injury detection as well as promotes the clinical application of machine learning systems.

## 4 Conclusion

This protocol paper outlined the significance and methodologically details of a systematic review of machine-learning-assisted detection for ACL injury. This systematic review and meta-analysis will provide high-quality synthesis of current evidence of machine learning system for detecting ACL injury based on MRI.

## Author contributions

Liang Yao and Peijing Yan contributed to study concept and design, Yongfeng Lao and Bibo Jia wrote the first draft and other authors had gave some suggestions for modification.

## Supplementary Material

Supplemental Digital Content
