# Supplementary material for: Diagnostic accuracy of machine-learning-assisted detection for anterior cruciate ligament injury based on magnetic resonance imaging: Protocol for a systematic review and meta-analysis
Source: Medicine (Baltimore). 2019 Dec 16;98(50):e18324. doi: 10.1097/MD.0000000000018324 (PMC6922500; doi:10.1097/MD.0000000000018324)
Supplement: Supplemental Digital Content [file medi-98-e18324-s001.docx]

Appendix 1. Search strategies in different databases

**PubMed:**

#1 "Artificial Intelligence"[MeSH]

#2 "Deep Learning"[MeSH]

#3 "Machine Learning"[MeSH]

#4 ((((((((Artificial intelligen*[Title/Abstract]) OR artificial intellect[Title/Abstract]) OR Machine Intelligence[Title/Abstract]) OR Computational Intelligence[Title/Abstract]) OR AI[Title/Abstract]) OR deep learning*[Title/Abstract]) OR machine learning*[Title/Abstract]))

#5 #1 OR #2 OR #3 OR #4

#6 "Anterior Cruciate Ligament Injuries"[MeSH]

#7 "Anterior Cruciate Ligament"[MeSH]

#8 (((((anterior cruciate ligament[Title/Abstract]) OR ACL[Title/Abstract]) OR anterior cruciate ligament injur*[Title/Abstract]) OR anterior cruciate ligament tear*[Title/Abstract]))

#9 #6 OR #7 OR #8

#10 #5 AND #9

**Cochrane Library:**

#1 MeSH descriptor: [Artificial Intelligence] explode all trees

#2 MeSH descriptor: [Deep Learning] explode all trees

#3 MeSH descriptor: [Machine Learning] explode all trees

#4 (Artificial intelligen*):ti,ab,kw OR (artificial intellect):ti,ab,kw OR (Machine Intelligence):ti,ab,kw OR (Computational Intelligence):ti,ab,kw OR (AI):ti,ab,kw

#5 (deep learning*):ti,ab,kw OR (machine learning*):ti,ab,kw

#6 #1 OR #2 OR #3 OR #4 OR #5

#7 MeSH descriptor: [Anterior Cruciate Ligament Injuries] explode all trees

#8 MeSH descriptor: [Anterior Cruciate Ligament] explode all trees

#9 (anterior cruciate ligament):ti,ab,kw OR (ACL):ti,ab,kw OR (anterior cruciate ligament injur*):ti,ab,kw OR (anterior cruciate ligament tear*):ti,ab,kw

#10 #7 OR #8 OR #9

#11 #6 AND #10

**EMBASE:**

#1 'deep learning'/exp OR 'artificial intelligence'/exp OR 'computational intelligence'/exp OR 'machine learning'/exp

#2 'artificial intelligence':ab,ti OR 'artificial intellect':ab,ti OR 'machine intelligence':ab,ti OR 'computational intelligence':ab,ti OR ai:ab,ti OR 'deep learning':ab,ti OR 'artificial intelligent':ab,ti OR 'machine learning':ab,ti

#3 #1 OR #2

#4 'anterior cruciate ligament'/exp OR 'anterior cruciate ligament injury'/exp OR 'anterior cruciate ligament tear'/exp

#5 'anterior cruciate ligament tear':ab,ti OR 'anterior cruciate ligament tears':ab,ti OR acl:ab,ti OR 'anterior cruciate ligament injury':ab,ti OR 'anterior cruciate ligament injuries':ab,ti OR 'anterior cruciate ligament':ab,ti

#6 #4 OR #5

#7 #3 AND #6

**Web of Science:**

# 1 TS=(Artificial intelligen* OR artificial intellect OR Machine Intelligence OR Computational Intelligence OR AI OR deep learning* OR machine learning*)

# 2 TS=(anterior cruciate ligament OR ACL OR anterior cruciate ligament injur* OR anterior cruciate ligament tear*)

# 3 #2 AND #1
